# Nondenaturing Purification of Co-Transcriptionally Folded RNA Avoids Common Folding Heterogeneity

**DOI:** 10.1371/journal.pone.0012953

**Published:** 2010-09-23

**Authors:** Miguel J. B. Pereira, Vivek Behera, Nils G. Walter

**Affiliations:** Single Molecule Analysis Group, Department of Chemistry, University of Michigan, Ann Arbor, Michigan, United States of America; University Paris 7, France

## Abstract

Due to the energetic frustration of RNA folding, tertiary structured RNA is typically characterized by a rugged folding free energy landscape where deep kinetic barriers separate numerous misfolded states from one or more native states. While most *in vitro* studies of RNA rely on (re)folding chemically and/or enzymatically synthesized RNA in its entirety, which frequently leads into kinetic traps, nature reduces the complexity of the RNA folding problem by segmental, co-transcriptional folding starting from the 5′ end. We here have developed a simplified, general, nondenaturing purification protocol for RNA to ask whether avoiding denaturation of a co-transcriptionally folded RNA can reduce commonly observed *in vitro* folding heterogeneity. Our protocol bypasses the need for large-scale auxiliary protein purification and expensive chromatographic equipment and involves rapid affinity capture with magnetic beads and removal of chemical heterogeneity by cleavage of the target RNA from the beads using the ligand-induced *glmS* ribozyme. For two disparate model systems, the Varkud satellite (VS) and hepatitis delta virus (HDV) ribozymes, we achieve >95% conformational purity within one hour of enzymatic transcription, without the need for any folding chaperones. We further demonstrate that *in vitro* refolding introduces severe conformational heterogeneity into the natively-purified VS ribozyme but not into the compact, double-nested pseudoknot fold of the HDV ribozyme. We conclude that conformational heterogeneity in complex RNAs can be avoided by co-transcriptional folding followed by nondenaturing purification, providing rapid access to chemically and conformationally pure RNA for biologically relevant biochemical and biophysical studies.

## Introduction

Eukaryotic cells contain a vast and diverse array of functional, small and large, often tertiary structured non-coding RNAs [1], [2]. Biochemical and biophysical studies of an increasing number of these RNAs have been primarily performed *in vitro*, and the results are generally thought to offer valuable insights into RNA behavior *in vivo* [3]. Traditionally, RNA molecules generated by chemical and/or enzymatic means are denatured during purification and must then be refolded *in vitro* in their entirety [4]–[7]. Central to most *in vitro* study has therefore been the (often implicit) assumption that RNA molecules properly refold into the native tertiary structures found *in vivo*, which are produced by co-transcriptional folding [8], often with the aid of folding enzymes called chaperones [7], [9]–[11]. Recently, evidence has accumulated for the existence of multiple, stable, functionally either active (native) or inactive (nonnative, misfolded) states of RNAs when refolded *in vitro* [12]–[16]. These results give renewed urgency to the still open question of whether refolding an entire RNA *in vitro* is a generally acceptable replacement for the segmental folding that occurs during transcription [7].

Several methods have been developed in recent years to purify RNA while maintaining its co-transcriptionally formed structure [17]–[22]. It is still unclear, however, whether these nondenaturing (or native) purification methods yield significantly different RNA folds in comparison to denaturing purification methods. For example, a recent study found no significant differences between natively- and non-natively purified group I intron variants, except for a somewhat higher propensity to crystallize [23]. We therefore sought to address this question for two disparate model systems from the class of small ribozymes, the hepatitis delta virus (HDV) and Varkud Satellite (VS) ribozymes. Whereas the HDV ribozyme is a tight, double-nested pseudoknot that has been repeatedly crystallized [24]–[27], a high-resolution crystal structure of the larger VS ribozyme has so far proven elusive [28]; these observations are consistent with the comparably high conformational heterogeneity and dynamics observed in single molecule folding studies of the VS ribozyme [29].

Shortcomings in previous native purification methods necessitated the development of a simplified and generalizable nondenaturing purification methodology. Our method purifies co-transcriptionally folded RNAs with homogenous 3′ ends to >95% conformational purity and avoids both denaturation and large-scale auxiliary protein purification. We use this method to demonstrate that refolding *in vitro* introduces severe conformational heterogeneity into the natively-purified Varkud Satellite (VS) ribozyme. By contrast, heterogeneity is not observed upon refolding of the compact double-nested pseudoknot of the hepatitis delta virus (HDV) ribozyme. We conclude that, depending on the particular RNA of interest, significant differences can exist between natively- and non-natively purified RNA populations. Our nondenaturing purification approach therefore paves the way for biologically relevant biochemical and biophysical studies of the vast array of emergent, often structurally complex, non-coding RNAs.

## Results

### Description of the purification protocol

**Figure 1:**
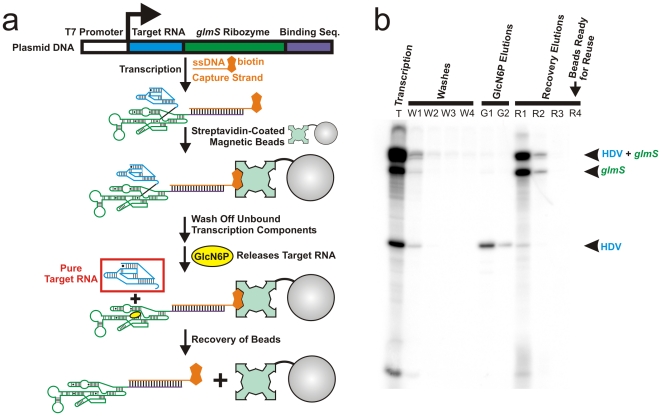
Native purification strategy and results. (**a**) Diagram of the strategy used to purify natively folded RNA. (**b**) Supernatants collected at various steps of the protocol and analyzed by denaturing, 8 M urea, 10% PAGE. “HDV” refers to the targeted self-cleaved HDV ribozyme, “*glmS*” to the self-cleaved *glmS* ribozyme with attached binding sequence, and “HDV+*glmS*” to the transcript containing the HDV ribozyme with attached uncleaved *glmS* ribozyme and binding sequence.

A schematic of our purification protocol is found in **Figure 1a**. Recombinant methods can be used to insert the DNA sequence encoding any target RNA of interest into the plasmid (available upon request). The target RNA gene lies immediately downstream of a T7 RNA polymerase (RNAP) promoter and upstream of the gene for the ligand-induced self-cleaving *glmS* ribozyme [20], [30], [31] as well as of a binding sequence used for bead capture. *In vitro* transcription using RNAP yields a transcript containing the target RNA fused to the *glmS* ribozyme and the binding sequence. The transcription reaction (**Figure 1b**, lane T) contains a biotinylated single-stranded (ss)DNA capture strand that forms a 20-base pair duplex with the binding sequence. After transcription, streptavidin-coated magnetic beads are added to capture the RNA:ssDNA hybrid on a solid support at room temperature, facilitating subsequent sample handling since supernatant is easily removed after applying a magnet to one side of the tube. Following removal of the supernatant, repeated wash steps purge all unwanted transcription components, including any abortive transcripts that lack at least part of the last-transcribed 3′ binding sequence (**Figure 1b**, lanes W1–W4). Addition of glucosamine-6-phosphate (GlcN6P) induces site-specific self-cleavage of the *glmS* ribozyme immediately 3′ to the target RNA (**Figure S1**), releasing the co-transcriptionally folded target RNA with homogeneous 3′ end from the solid support (**Figure 1b**, lanes G1–G2). After collecting the target RNA within an hour of completion of the transcription reaction, the strong biotin-streptavidin interaction can be broken [32] to recover the solid support for further use (**Figure 1b**, lanes R1–R4).

Application of this protocol to an 78-nucleotide (nt) antigenomic HDV ribozyme associated with the human pathogen HDV [33] (**Figure S1**) leads to collection of self-cleaved (and thus active) target ribozyme at >99% purity (as judged by denaturing polyacrylamide gel electrophoresis (PAGE); **Figure 1b**, lanes G1–G2). In addition, lanes R3–R4 in **Figure 1b** demonstrate that the magnetic beads are subsequently depleted of any remaining trace of the RNA:ssDNA hybrid, and thus are ready to be reused.

### Optimization of the purification

**Figure 2:**
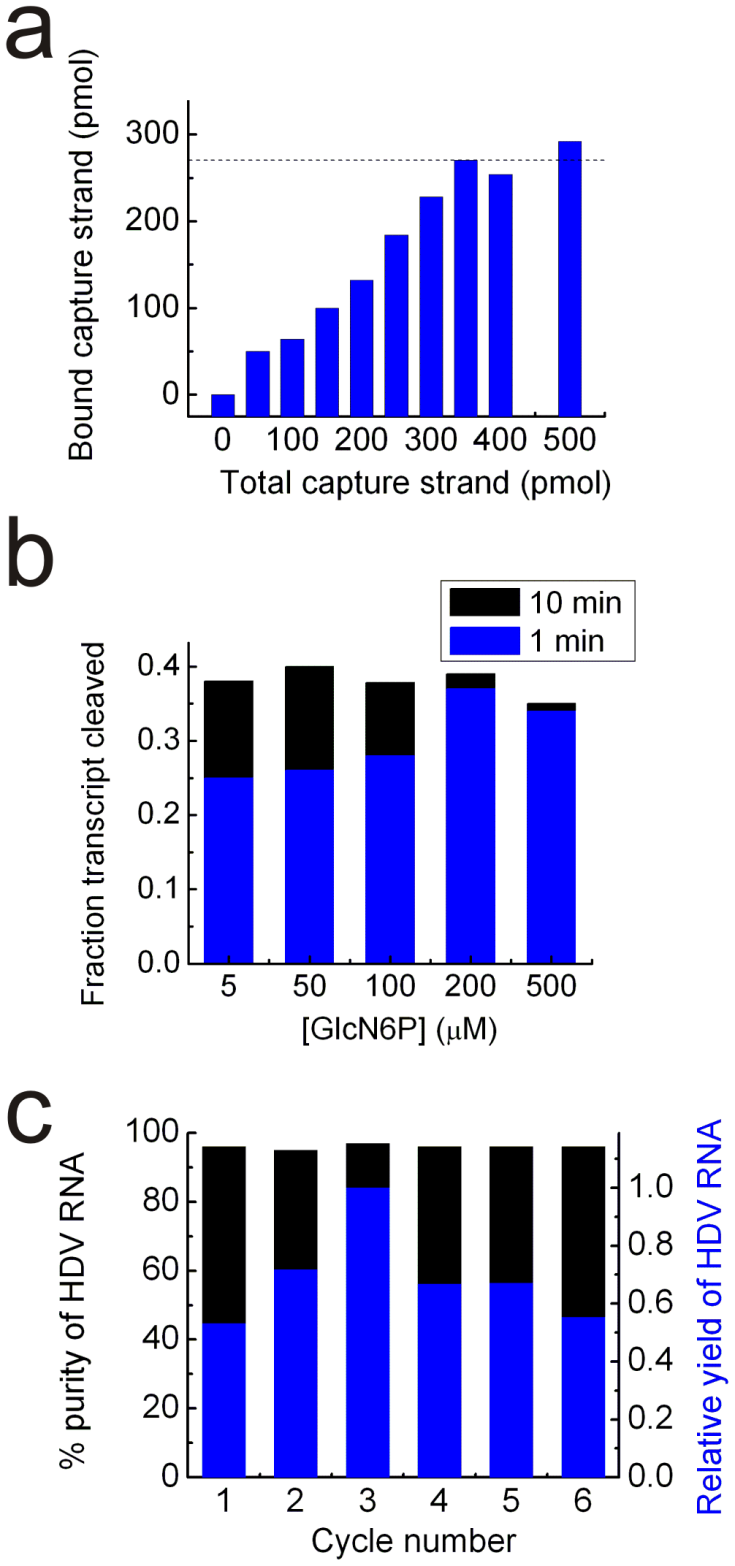
Optimization of important protocol parameters using the transcript containing HDV ribozyme, *glmS* ribozyme, and binding sequence. (**a**) Titration of 100 µl completed transcription reaction with ssDNA capture strand as analyzed by electrophoretic mobility shift assay (EMSA). The dotted line indicates where we find the amount of RNA:ssDNA hybrid to level off. (**b**) Characterization of *glmS* ribozyme self-cleavage kinetics. Aliquots of cleavage reaction mix at varying GlcN6P concentrations were taken over a range of times and analyzed by denaturing PAGE. Only the 1 and 10 min time points are shown. (**c**) Streptavidin-coated magnetic beads do not deteriorate over multiple cycles of use. The percent purity of HDV ribozyme as judged by denaturing PAGE analysis is plotted on the left axis, whereas the relative yield of HDV ribozyme (normalized to cycle 3) is plotted on the right axis.

First, excess capture strand will compete for the streptavidin available on the magnetic beads, while using too little capture strand will prevent some transcribed RNA from attaching to the beads. We therefore varied the amount of capture strand and found that ∼350 picomoles of capture strand was just about saturating (**Figures 2a** **and S2**) when included in the 100-µl transcription reaction; this amount has to be optimized on a case-by-case basis. Exactly saturating the RNA with capture strand will maximize the RNA yield since any additional capture strand will not bind RNA but compete with the RNA-bound capture strand for binding sites on the streptavidin-coated beads. Second, robust activity of the *glmS* ribozyme is critical to the release of target RNA. We therefore measured the extent of *glmS* ribozyme cleavage over time at varying concentrations of GlcN6P (**Figures 2b** **and S3**). The maximal extent of cleavage (which is ∼40%) requires only 200 µM GlcN6P and an incubation time of ∼1 min (**Figure S3b**). The same cleavage extent can be achieved at a 40-fold lower concentration of 5 µM GlcN6P if the reaction proceeds for 10 min (**Figure 2b**). For our standard protocol we chose the former conditions. Third, economical application of our purification method requires that the streptavidin-coated beads be reused for multiple cycles of RNA purification. To this end, the same sample of beads was used to collect HDV RNA over six successive applications of the protocol, including complete removal of the biotinylated capture strand (**Figures 2c** **and S4**). HDV ribozyme purity was maintained at >96% throughout and no significant decrease was observed in the amount of RNA purified per cycle.

### Nondenaturing purification of co-transcriptionally folded RNA avoids refolding heterogeneity

**Figure 3:**
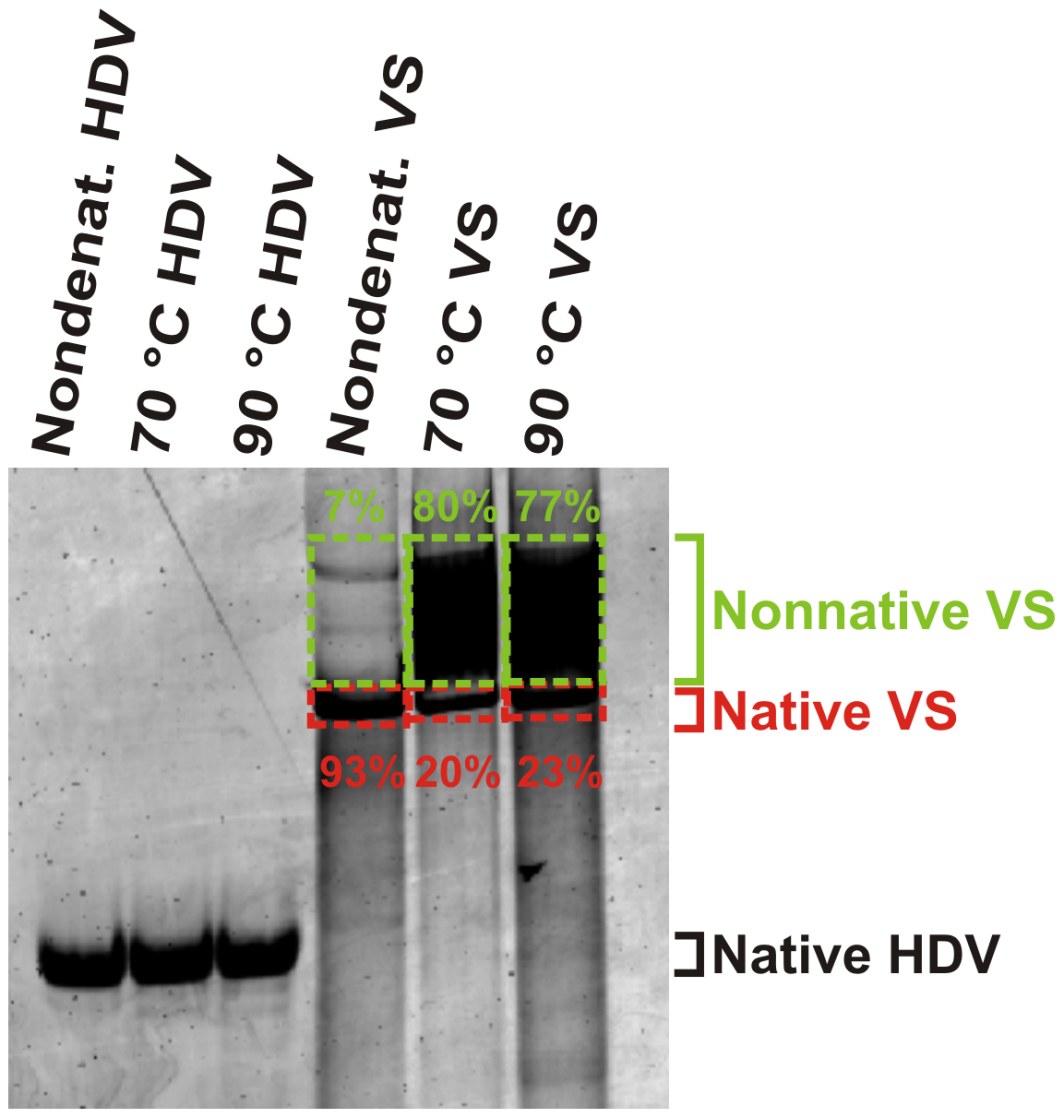
Heat annealing (refolding) introduces RNA structures not found upon native purification. Samples were analyzed by EMSA using non-denaturing PAGE. Samples in lanes 1 and 4 were purified using our native protocol. Lanes 2, 3, 5, and 6 are samples of natively purified RNA that were subsequently heat annealed at the indicated temperatures. The boxed bands with percentages in green represent the fractions of VS ribozyme found in a single (as judged by EMSA), high-mobility, native (as judged by the fact that this fraction is self-cleaved) conformation. The boxed smears with percentages in red represent the fractions of VS ribozyme found in alternative, lower-mobility, heterogeneous conformations.

Development of this native purification protocol allowed us to test the impact of heat denaturation and refolding on co-transcriptionally folded RNA. We examined this effect on two RNAs of varying sizes: the 78-nt HDV ribozyme as discussed above, as well as the 164-nt Varkud Satellite (VS) ribozyme (based on the well-studied G11 construct [29], [34]), which was also produced to >99% purity (**Figure S5a,b**). Samples of natively purified, self-cleaved HDV and VS ribozymes were heated to either 70°C or 90°C, annealed by slow cooling to room temperature, then analyzed by non-denaturing electrophoretic mobility shift assay (EMSA) in comparison with the unheated RNA. The RNA concentrations were ∼0.5 µM, in accordance with a study showing that concentrations below 3 µM more effectively regenerate RNA secondary structures during heat annealing [35]. Strikingly, heat annealing had no significant impact on the HDV ribozyme, but a profound effect on the VS ribozyme, where refolding resulted in >70% of the RNA forming alternative, more slowly migrating (i.e., less tightly folded) conformations not found in the never-denatured, self-cleaved and thus catalytically active sample (**Figure 3**). That is, even heat annealing under conditions most typically used for producing well-folded RNA yielded conformationally much more heterogeneous VS ribozyme than our rapid nondenaturing purification protocol.

## Discussion

In this study, we report the first instance of a ribozyme that demonstrably folds differently upon nondenaturing and denaturing purification. It was previously observed that the large (246–341 nt) group I introns showed no structural differences upon artificial refolding [24], a result we also found true for the small (78 nt) HDV ribozyme. The size of an RNA is not, however, an accurate predictor of its ability to properly refold after denaturation. The heating and subsequent cooling of an RNA of intermediate size, the VS ribozyme of 164 nt length, resulted in the conversion of most of the native fold(s) into slower migrating non-native folds (**Figure 3**). The appearance of these non-native isomers upon complete refolding correlates with the VS ribozyme's incomplete cleavage and heterogeneous equilibrium folding dynamics at the single molecule level [29] and may explain the many failed attempts to crystallize it [28]. These results imply that, although some RNA folds tolerate (partially) denaturating conditions, the generation of a conformationally homogeneous population is more generally achieved by native purification of co-transcriptionally folded RNA. The difficulty of predicting whether a given RNA can successfully refold after denaturation necessitates the use of native purification for subsequent biochemical and/or biophysical characterization.

Several protocols for native purification of RNA have been previously described [17]–[22]. A precursor method to the one described here utilizes affinity capture on a Ni^2+^-agarose chromatography column and subsequent self-cleavage of the affinity tag [20]. While successful in generating native RNA transcripts with homogenous 3′ ends, this method requires the user to FPLC-purify large quantities of an auxiliary His-tagged MBP-MS2 coat fusion protein. Other procedures have bypassed the need for large-scale protein purification by employing gel filtration chromatography [21]. Such methods, however, suffer from limited resolution power, RNA transcript 3′ heterogeneity, and time-consuming preparative purification steps. A more recent method bypasses the need for these preparative steps by using anion-exchange chromatography to separate large-scale RNA transcription products on an FPLC column. This method does not, however, eliminate either RNA transcript 3′ heterogeneity or the need for an RNA-dedicated FPLC system. The drawbacks of these methods motivated the design of a more practical, rapid, and efficient way to natively purify RNA.

We describe here a protocol that achieves >95% chemical and conformational purity within an hour of transcription for two disparate model RNAs. The chemical purity, as evident from the observation of a single band in lanes G1–G2 compared to the other lanes of **Figure 1b** (see also **Figure 3**), requires a strong binding motif. The 20-bp duplex formed between the capture strand and the RNA transcript stably anchors the target RNA to the solid support matrix, while avoiding the need for an auxiliary binding protein [18], [21]. This feature together with the use of magnetic beads allows for rapid supernatant removal and the necessary washing steps that follow. It also allows for the specific release of the target RNA within 1 min upon addition of GlcN6P to induce self-cleavage of the embedded ligand-induced *glmS* ribozyme. Site-specific self-cleavage results in a target RNA that is chemically pure in terms of its 3′ end homogeneity. Overall, our method only requires the purchase of commercially available magnetic beads, the accompanying magnet, and standard biochemical tools for *in vitro* transcription.

Our method is straightforward to scale up, allowing for a wide range of applications. The protocol was developed on a scale that yields ∼50 picomoles of pure target RNA per 100 µl transcription reaction. We have also scaled it up to yield ∼500 picomoles of target RNA from a 1-ml transcription reaction (data not shown), and the necessary equipment is readily available for scaling it up to a 100-ml transcription reaction to yield ∼50 nanomoles of nondenatured target RNA. Several rounds of purification using the same magnetic beads would yield enough (∼500 nanomoles) RNA for high-resolution structural characterization by X-ray crystallography or NMR.

Our methodology was here successfully exploited to co-transcriptionally purify two different catalytic RNAs, and the plasmid used in this procedure (**Figure** 1) allows for application to any RNA. The plasmid was adapted for general use by incorporation of a universal multiple cloning site, into which any target RNA gene can be inserted (**Figure S6a**). An alternate version of this plasmid that contains an inactive T7 promoter (**Figure S6b**) is also available upon request and is intended to be used in case an RNA of a specific 5′ sequence is desired, which will require cloning of a T7 promoter immediately upstream of the RNA gene. There is no requirement for the target RNA to be catalytically active or to have enhanced folding when transcribed in proximity to the glmS ribozyme. The only role of the glmS ribozyme in this procedure is to specifically select for the target RNA by GlcN6P-dependent elution. Since denaturation is entirely avoided, the purified RNA sample will in turn reflect the distribution of structures formed during transcription.

We did not investigate here the precise mechanism of co-transcriptional folding and why it would yield in case of the VS ribozyme a different fold than heat denaturation followed by reannealing. Wong et al. have suggested that nature utilizes transcriptional pause sites between the upstream and downstream segments of native long-range helices to kinetically guide their formation through the transient adoption of labile nonnative structures [8]. The Varkud satellite RNA harboring the VS ribozyme is found in mitochondria of the bread mold *Neurospora* [36], [37] and is thus transcribed by a eukaryotic RNA polymerase rather than a bacteriophage enzyme such as the T7 RNA polymerase used in our experiments. Our finding that segmental co-transcriptional folding has a profound impact on folding of the VS ribozyme during *in vitro* transcription thus suggests that some general principles are at work among diverse RNA polymerases under varying conditions.

In conclusion, we have developed an improved nondenaturing purification protocol that demonstrates significant conformational differences between natively- and non-natively purified RNA. We show conclusively that purification of RNA co-transcriptionally folded *in vitro* avoids folding heterogeneities otherwise observed in RNA that is heat denatured and subsequently refolded in its entirety, as is currently the standard protocol in the field. Apparently, segmental folding from the 5′ end during transcription yields a fundamentally different conformational ensemble for certain RNAs than denaturation/refolding, even in the absence of any chaperones postulated to play an important role for RNA folding *in vivo* [11], [38]. These results are not unexpected given the energetically frustrated nature of the RNA folding process, caused by RNA's relatively small number of chemical building blocks compared to that of proteins [9], [39]. Such frustrated folding leads to a rugged free energy landscape *in vitro*, featuring multiple local and global minima that can represent either functionally active or inactive states [12]–[16]. Our results suggest that segmental folding during transcription safely guides an RNA across the rugged folding free energy landscape towards the native state(s) in a way that heat annealing does not (we note that both the HDV and VS ribozyme have self-cleaved during transcription, attesting to the fact that they are both natively folded). This finding strongly argues for a more widespread use of native purification protocols in RNA research. In addition, the critical question can finally be addressed of what causes refolding heterogeneity commonly observed upon denaturation of non-coding RNAs, so many of which have recently been discovered to be of central importance in the biology of eukaryotes [1], [2].

## Materials and Methods

### Generation of plasmids for *in vitro* transcription

The pVS plasmid encodes for the VS ribozyme. A PCR product was generated using a stepwise preparation and purification procedure based on four nested primers with 5′ overhangs and the plasmid topWT [29], digested with EcoRI and HindIII, then inserted into the multiple cloning site of the equally doubly-digested pGEM4Z plasmid (Promega). The insert contains an AflII site embedded nine nucleotides from the start of the *glmS* ribozyme gene, and a U1A-protein binding site [40] in the VS ribozyme sequence (**Figure S5**).

The pVS plasmid served as parental plasmid for the construction of the HDV ribozyme encoding plasmid pHDV. The pVS plasmid was simultaneously digested with HindIII and AflII, the enzymes heat inactivated at 70°C for 20 min, the DNA phenol chloroform extracted, and the product cut out after electrophoresis on a 1% agarose gel using a commercial extraction kit (Qiagen, catalog no. 28706). A PCR product (prepared by extension of two partially overlapping primers) was simultaneously digested with HindIII and AflII, the enzymes heat inactivated at 70°C for 20 min, and the DNA phenol chloroform extracted prior to insertion into the prepared doubly digested pVS plasmid using T4 DNA ligase as suggested by the manufacturer (Invitrogen) to generate the pHDV plasmid.

The same HindIII and AflII digested pVS plasmid was used to generate pMCGL through inserting into the overlap region two synthetic, partially complementary oligodeoxynucleotides with sticky 5′ phosphorylated ends and the multiple cloning site sequence (**Figure S6**). The pMCGLΔT7 plasmid (**Figure S6**) was generated through site-directed mutagenesis of pMCGL. DNA sequencing was used to confirm all plasmid sequences. All synthetic DNA oligonucleotides used in this study were purchased from Invitrogen and purified by denaturing PAGE.

### Native Purification of co-transcriptionally folded HDV and VS ribozymes

A 150-µl transcription reaction with 4 mM of each NTP, 40 mM HEPES-KOH, pH 8.0, 5 mM DTT, 25 mM MgCl_2_, 200 mM NaCl, 50 nM EcoRI-linearized pHDV or pVS plasmid, 0.008 U/µl inorganic pyrophosphatase, 0.1 mg/ml T7 RNAP, 350 pmol capture strand, and trace amounts (10 µCi) of ^32^P-α-GTP was incubated at 37°C for 2 h. The capture strand oligonucleotide was biotinylated on the 5′ end and had the following sequence: 5′-AAAAAAAAAAGAATTCCGCAGGCCTGCTCG-3′. Phenol/chloroform extraction yielded ∼100 µl final volume (without this removal of RNAP the beads tended to stick to the side of the microcentrifuge tube once added), to which 25 µl of 5 M NaCl was added for a final Na^+^ concentration of ∼1 M. This reaction mixture was added to 100 µl (1 mg) of Streptavidin C1 Dynabeads (Invitrogen, catalog no. 650.01) equilibrated in wash buffer (WB: 40 mM HEPES-KOH, pH 7.4, 1 M NaCl).

This bead suspension was rotated at room temperature for 30 min to form the complex between the biotinylated RNA:ssDNA hybrid and the streptavidin-coated magnetic beads. A magnetic particle concentrator (MPC) was applied to remove the supernatant and yield a solid bead pellet (analyzed in lane “T” in **Figure 1b**). The beads were then washed with 100 µl of WB, and the supernatant was collected after applying the MPC. A wash step entails the resuspension of the concentrated beads with WB, followed by removal of buffer through the use of the MPC. This WB wash was performed a total of four times (lanes “W1–W4” in **Figure 1b**).

The target RNA was eluted by the addition of 100 µl cleaving buffer (CB: 40 mM HEPES-KOH, pH 7.4, 1 M NaCl, 10 mM MgCl_2_, 200 µM GlcN6P) to the bead pellet. The sample was incubated for 1 min, upon which the supernatant was collected. This CB wash was performed a total of twice (lanes “G1–G2” in **Figure 1b**).

The beads were then washed with 100 µl of elution buffer (EB: 10 mM Tris, pH 8.0). This EB wash was performed a total of three times, and the supernatants were discarded. The EB wash was then performed a 4^th^ time, followed by incubation in a 70°C water bath for 5 min, incubation at room temperature for 5 min, and collection of the supernatant. This EB wash with heating was performed a total of twice (lanes “R1–R2” in **Figure 1b**). Two more EB washes were performed without heating (lanes “R3–R4” in **Figure 1b**).

All collected supernatants were placed on the MPC a second time to remove any residual magnetic beads; a 20µl aliquot of each supernatant was removed and added to an equal volume of denaturing loading buffer (80% (v/v) formamide, 0.025% (w/v) xylenecyanol blue, 0.025% (w/v) bromophenol blue and 10 mM EDTA) and analyzed by denaturing, 8 M urea, 10% PAGE, run in 1× TBE at 20 W for 2 h. The gel was exposed to a PhosphorImager screen overnight and quantified using a Typhoon 9410 Variable Mode Imager with ImageQuant software (GE Healthcare). The band intensities were corrected for the number of guanines in each sequence so that the intensity values report on the relative counts of RNA molecules within each band. These counts were used to calculate the fractional purities of specific molecule populations detected in the supernatants.

### Activity assay of the *glmS* ribozyme

Self-cleavage activity of the glmS ribozyme was assayed following the native purification protocol described above, with slight modifications only to the cleavage step. The CB added to the beads was composed of 40 mM HEPES-KOH, pH 7.4, 1 M NaCl, 10 mM MgCl_2_, and GlcN6P concentrations of 5, 50, 100, 200 or 500 µM. Progression of the reaction was monitored by removing 10 µl aliquots that included the suspended beads after 0.25, 0.5, 0.75, 1, 2, 5, and 10 min and mixing them with 30 µl of stop buffer (SB: 90% (v/v) formamide in 1× TBE). The different RNA species present were separated by denaturing, 8 M urea, 10% PAGE, run in 1× TBE at 20 W for 2 h. The zero time point was taken just prior to removal of WB so that the CB buffer could be added to the beads to initiate the reaction. The gel again was exposed overnight to a PhosphorImager screen, the intensities of the HDV ribozyme band were quantified using a Typhoon 9410 Variable Mode Imager with ImageQuant software (GE Healthcare) and corrected for the number of guanines as described above. The fraction cleaved was then calculated as H_counts_/(H_counts_+HG_counts_), where H represents the self-cleaved HDV ribozyme species and HG represents the HDV+*glmS* ribozyme species. The 200 µM GlcN6P data (**Figure S3b**) were fit with the single-exponential first-order rate equation 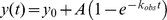 to yield the maximally cleaved fraction A and the observed rate constant k_obs_.

### Capture strand binding assay analyzed by EMSA

In ten independent 50-µl transcription reactions varying amounts of ssDNA capture strand (0, 25, 50, 75, 100, 125, 150, 175, 200, and 250 pmol) were included. Control reactions (lanes “200” and “500” in gel inset of **Figure S2**) consisted of transcriptions with 100 or 250 pmol capture strand prepared identically as above except that the linearized pHDV transcription template was omitted. 5-µl aliquots were removed and analyzed by EMSA on a nondenaturing 6% PAGE in 1× TBE at 5 W for 4 h. The gel was stained using a 1∶100,000 dilution of SYBR Gold (Invitrogen, catalog no. S11494) in 1× TBE for 5 min. The fluorescence intensity in the gel was measured using a Typhoon 9410 Variable Mode Imager (GE Healthcare) with an excitation of 488 nm, an emission bandpass of 530±10 nm, and a PMT setting of 500 V. The results were quantified using ImageQuant software (GE Healthcare). The fluorescence intensities of the free capture strand were converted into pmol amounts using the 100 and 250 pmol control standards of free capture strand in side lanes of the gel. The calculated free picomoles of capture strand were subtracted from the known amounts included at the beginning of the transcription reaction to give the bound pmol of capture strand shown in **Figure 2a**. The numbers reported in **Figure 2a** were scaled by a factor of 2 to report the results relative to transcription volumes of 100 µl.

### Heat annealing assay analyzed by EMSA

Both co-transcriptionally folded HDV and VS ribozymes underwent our standard native purification procedure as described above. 10-µl aliquots of the lane G1 (**Figure 1b**) solution were removed and heated to either 70°C or 90°C for 2 min, then cooled at room temperature over 10 min analyzed by EMSA on a nondenaturing 10% PAGE in 1× TBE at 20 W for 2.5 h. SYBR Gold staining, measurement of fluorescence intensity, and quantification were carried out as described for the capture strand binding assay.

## Supporting Information

Figure S1 The HDV-glmS-binding sequence construct used in this study. The self-cleaving HDV ribozyme is indicated in grey (5′ sequence) and cyan (self-cleaved ribozyme; contains a U1A protein binding site in bold), the glmS ribozyme in shown in green, and restriction enzyme sites used for cloning the plasmid transcription template are highlighted in red. The sites of self-cleavage of the two ribozymes are indicated by arrows; the color coded legend describes the nucleotide (nt) lengths of the resulting self-cleaved transcript fragments, with that of the target RNA boxed. The binding sequence is the boxed segment at the 3′ end of the glmS ribozyme that forms a hybrid with the biotinylated ssDNA capture strand (orange).
(0.84 MB TIF)

Figure S2 Capture strand titration. Samples from the capture strand binding assay, including varying concentrations of ssDNA capture strand added to the transcription reaction as indicated, were analyzed by EMSA using non-denaturing 6% PAGE in 1× TBE, followed by SYBR Gold staining and visualization with a Typhoon 9410 Variable Mode Imager. Lanes 1 and 12 are control lanes representing 200 and 500 pmol of capture strand, respectively, that were used to calibrate the amount of free capture strand in the titration lanes.
(1.05 MB TIF)

Figure S3 Monitoring glmS ribozyme self-cleavage over time in the presence of 200 µM GlcN6P in CB at 25 oC. (a) Aliquots with the suspended beads were removed at various time points and analyzed by denaturing, 8 M urea, 10% PAGE and subsequent autoradiography. Increases in density of the glmS and HDV ribozyme populations over time indicate self-cleavage of the glmS ribozyme. (b) Plot of the fraction of the HDV+glmS band converted into the HDV band over time after correcting for the number of guanines in both sequences.
(0.28 MB TIF)

Figure S4 Regenerating streptavidin-coated magnetic beads for reuse. 20-µl aliquots from the 4th WB wash (W), the 1st CB elution (G, containing GclN6P), and the 1st removal elution with heated EB (R) were taken from each cycle and analyzed by denaturing 10% PAGE. The lower band is cleaved HDV ribozyme, while the upper bands are the HDV+glmS and self-cleaved glmS ribozyme species, respectively, as indicated.
(0.62 MB TIF)

Figure S5 Application of the purification protocol to the VS ribozyme. (a) The VS-glmS-binding sequence construct used in this study. The self-cleaving VS ribozyme is indicated in grey (5′ sequence) and cyan (self-cleaved ribozyme; contains a U1A protein binding site in bold), the glmS ribozyme in shown in green, and restriction enzyme sites used for cloning the plasmid transcription template are highlighted in red. The sites of self-cleavage of the two ribozymes are indicated by arrows; the color coded legend describes the nucleotide (nt) lengths of the resulting self-cleaved transcript fragments, with that of the target RNA boxed. The binding sequence is the boxed segment at the 3′ end of the glmS ribozyme that forms a hybrid with the biotinylated ssDNA capture strand (orange). (b) Supernatants collected at various steps of the protocol and analyzed by denaturing, 8 M urea, 10% PAGE. “VS” refers to the targeted self-cleaved VS ribozyme, “glmS” to the self-cleaved glmS ribozyme with attached binding sequence, and “VS+glmS” to the transcript containing the VS ribozyme with attached uncleaved glmS ribozyme and binding sequence.
(1.34 MB TIF)

Figure S6 Sequences of the available plasmids with multiple cloning sites. The nucleotides and dotted line in green denote the sequence that encodes the glmS ribozyme. (a) The pMCGL plasmid contains an active T7 promoter upstream of the multiple cloning site. (b) In the pMCGLΔT7 plasmid the T7 promoter is inactivated by four point mutations (cyan).
(0.61 MB TIF)
