# Supplementary figures and images for: Nondenaturing Purification of Co-Transcriptionally Folded RNA Avoids Common Folding Heterogeneity
Source: PLoS One. 2010 Sep 23;5(9):e12953. doi: 10.1371/journal.pone.0012953 (PMC2944885; doi:10.1371/journal.pone.0012953)

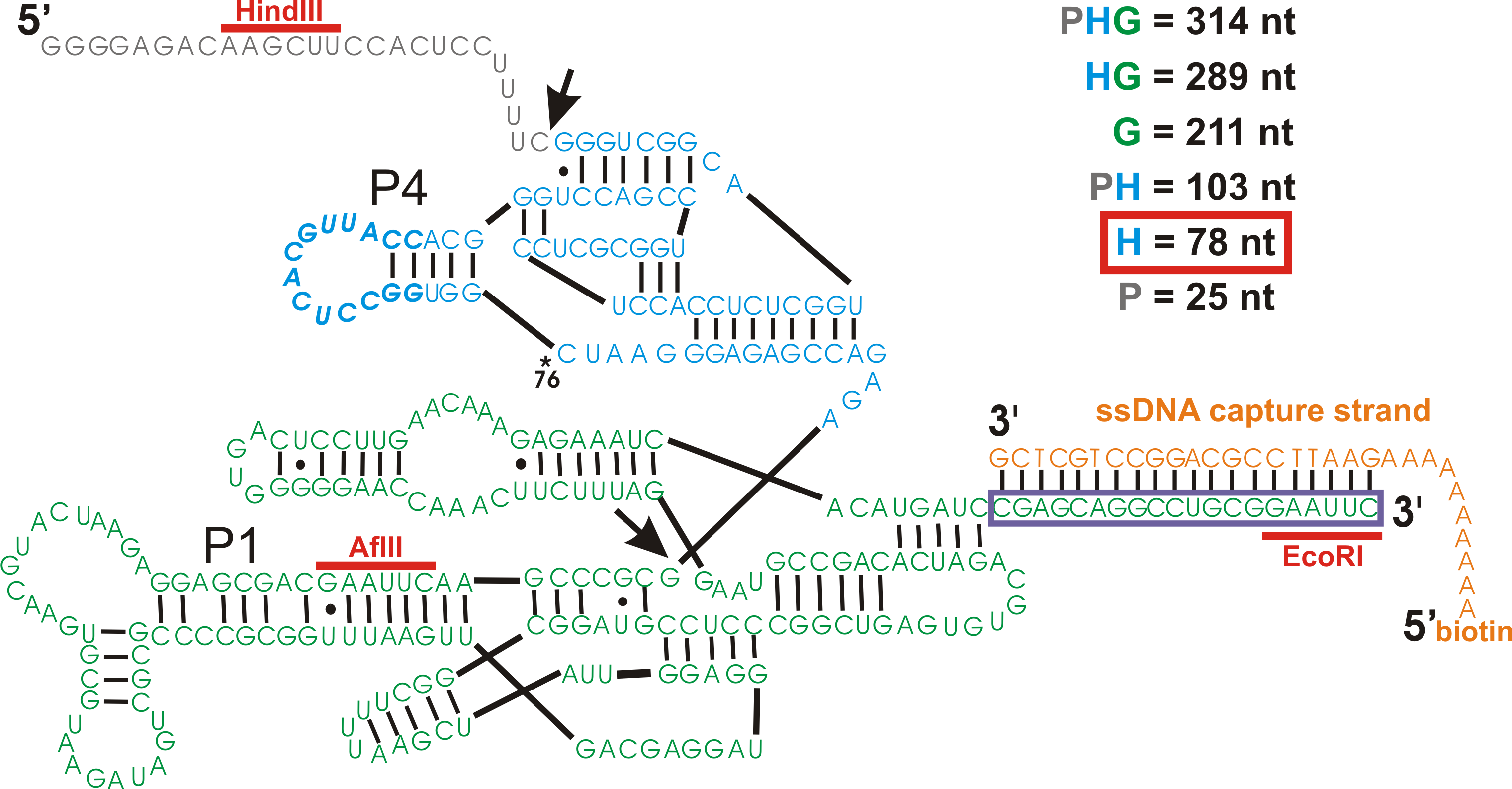

Supplement: Figure S1 — The HDV-glmS-binding sequence construct used in this study. The self-cleaving HDV ribozyme is indicated in grey (5′ sequence) and cyan (self-cleaved ribozyme; contains a U1A protein binding site in bold), the glmS ribozyme in shown in green, and restriction enzyme sites used for cloning the plasmid transcription template are highlighted in red. The sites of self-cleavage of the two ribozymes are indicated by arrows; the color coded legend describes the nucleotide (nt) lengths of the resulting self-cleaved transcript fragments, with that of the target RNA boxed. The binding sequence is the boxed segment at the 3′ end of the glmS ribozyme that forms a hybrid with the biotinylated ssDNA capture strand (orange). (0.84 MB TIF) [file pone.0012953.s001.tif]

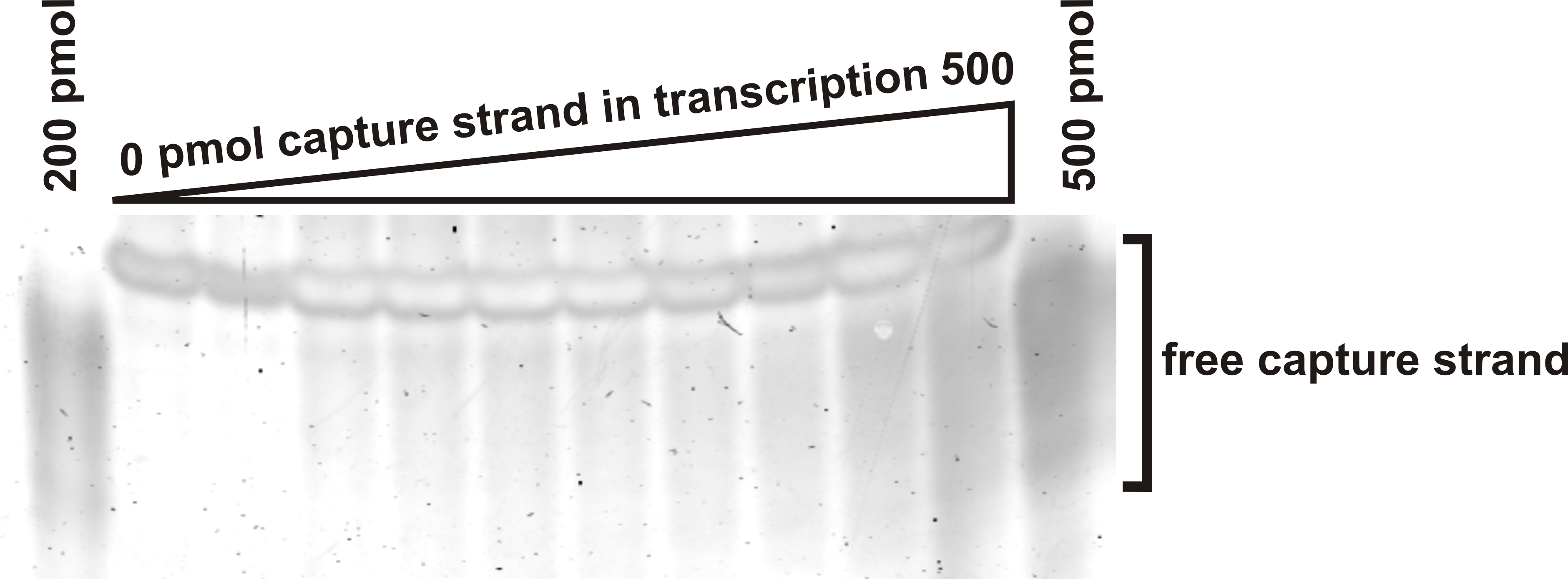

Supplement: Figure S2 — Capture strand titration. Samples from the capture strand binding assay, including varying concentrations of ssDNA capture strand added to the transcription reaction as indicated, were analyzed by EMSA using non-denaturing 6% PAGE in 1× TBE, followed by SYBR Gold staining and visualization with a Typhoon 9410 Variable Mode Imager. Lanes 1 and 12 are control lanes representing 200 and 500 pmol of capture strand, respectively, that were used to calibrate the amount of free capture strand in the titration lanes. (1.05 MB TIF) [file pone.0012953.s002.tif]

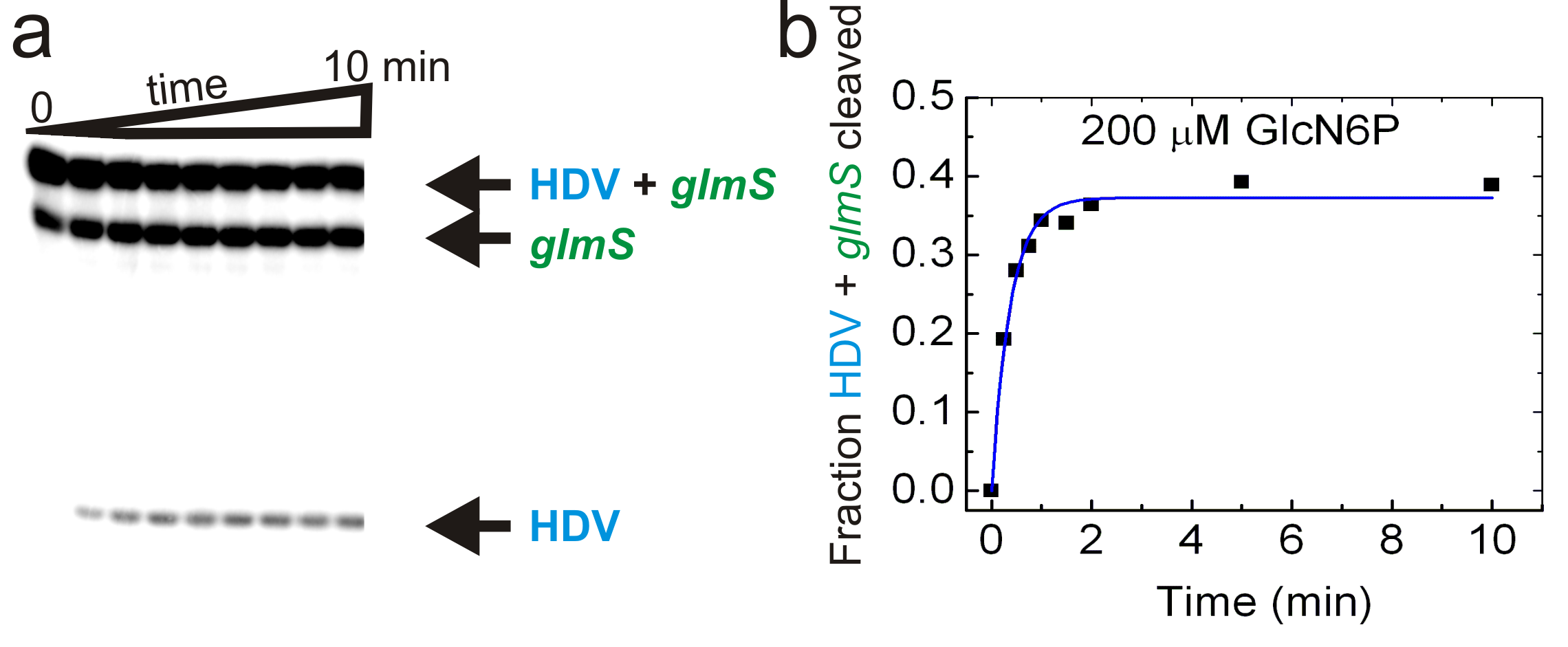

Supplement: Figure S3 — Monitoring glmS ribozyme self-cleavage over time in the presence of 200 µM GlcN6P in CB at 25 oC. (a) Aliquots with the suspended beads were removed at various time points and analyzed by denaturing, 8 M urea, 10% PAGE and subsequent autoradiography. Increases in density of the glmS and HDV ribozyme populations over time indicate self-cleavage of the glmS ribozyme. (b) Plot of the fraction of the HDV+glmS band converted into the HDV band over time after correcting for the number of guanines in both sequences. (0.28 MB TIF) [file pone.0012953.s003.tif]

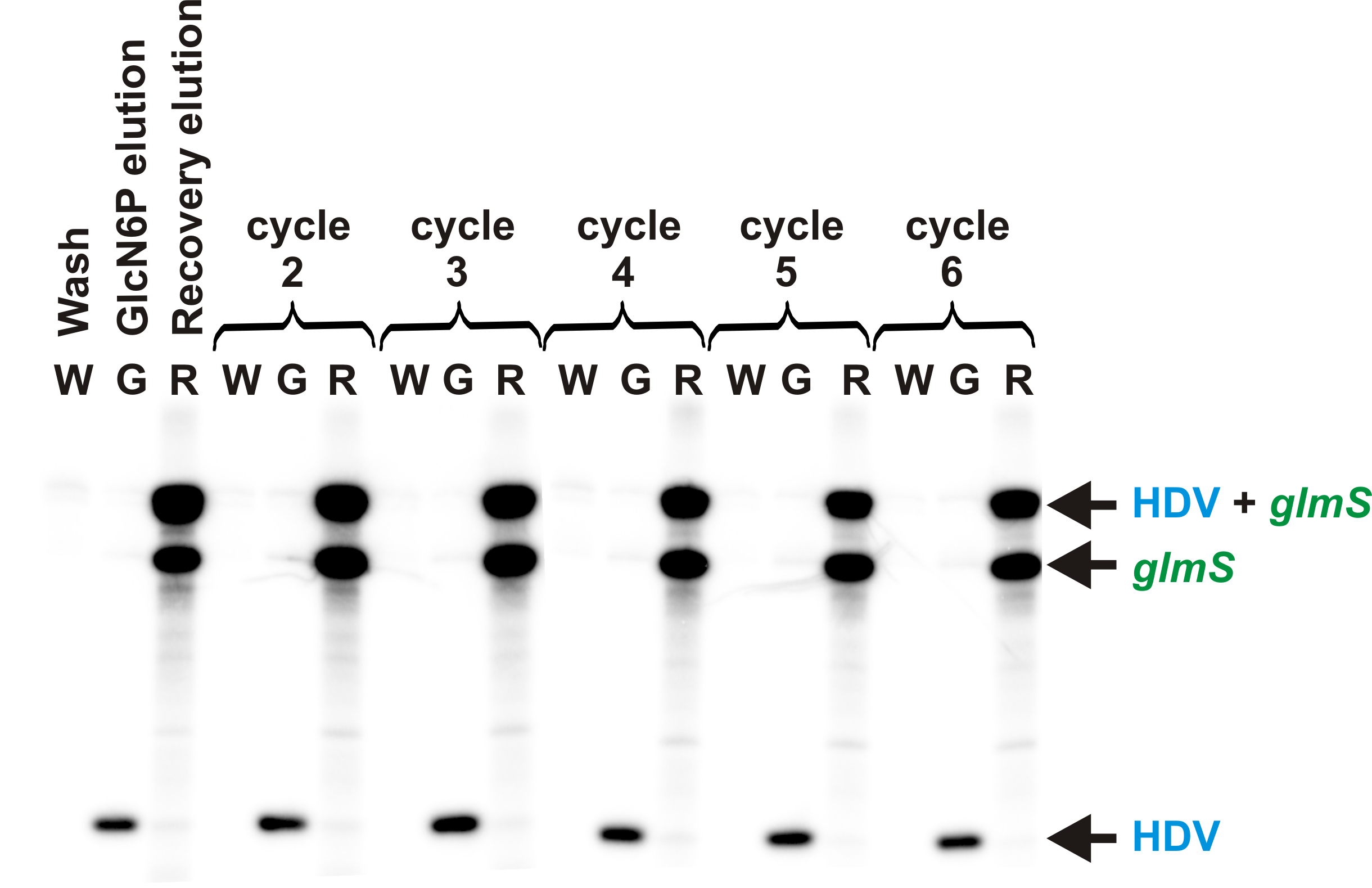

Supplement: Figure S4 — Regenerating streptavidin-coated magnetic beads for reuse. 20-µl aliquots from the 4th WB wash (W), the 1st CB elution (G, containing GclN6P), and the 1st removal elution with heated EB (R) were taken from each cycle and analyzed by denaturing 10% PAGE. The lower band is cleaved HDV ribozyme, while the upper bands are the HDV+glmS and self-cleaved glmS ribozyme species, respectively, as indicated. (0.62 MB TIF) [file pone.0012953.s004.tif]

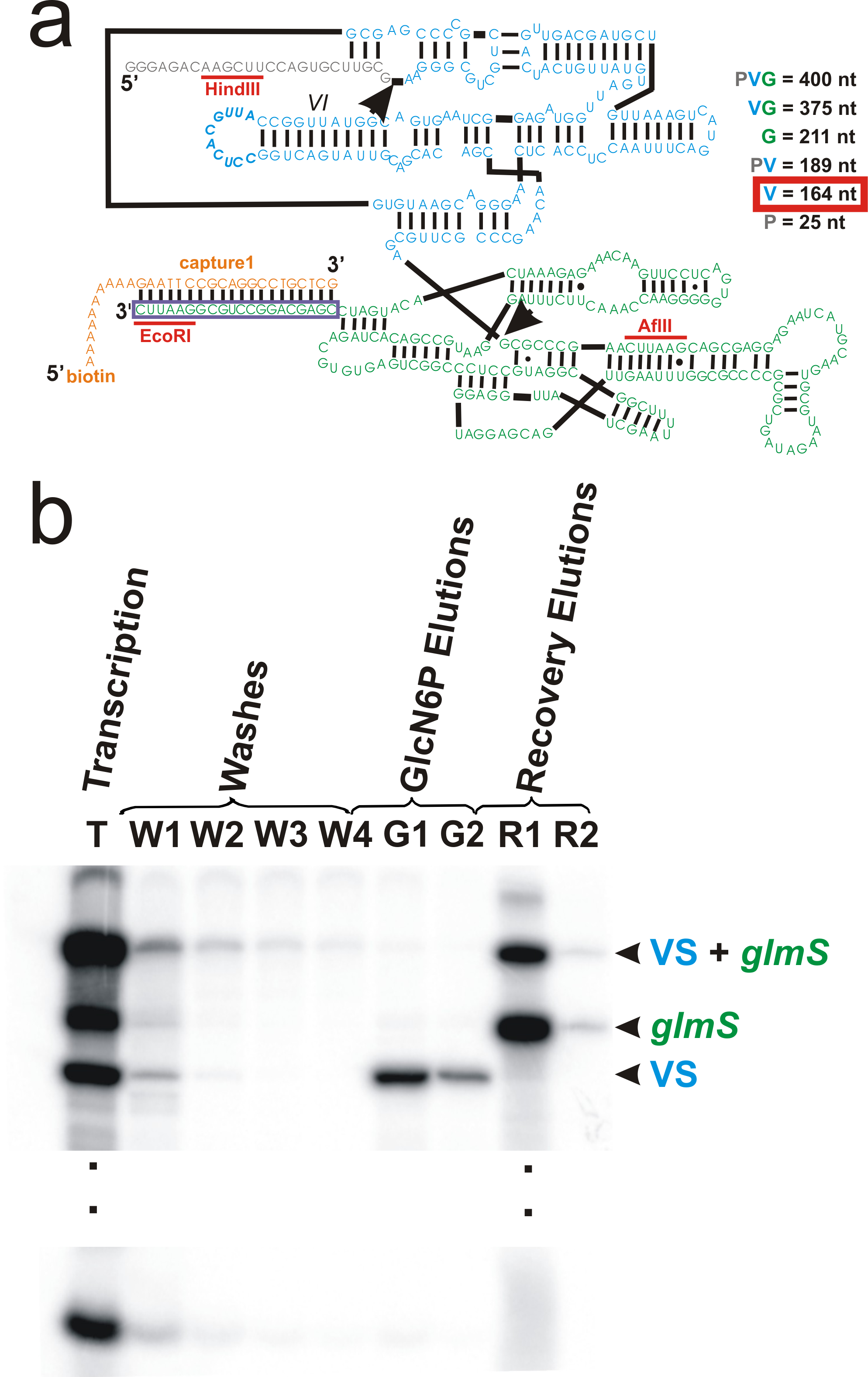

Supplement: Figure S5 — Application of the purification protocol to the VS ribozyme. (a) The VS-glmS-binding sequence construct used in this study. The self-cleaving VS ribozyme is indicated in grey (5′ sequence) and cyan (self-cleaved ribozyme; contains a U1A protein binding site in bold), the glmS ribozyme in shown in green, and restriction enzyme sites used for cloning the plasmid transcription template are highlighted in red. The sites of self-cleavage of the two ribozymes are indicated by arrows; the color coded legend describes the nucleotide (nt) lengths of the resulting self-cleaved transcript fragments, with that of the target RNA boxed. The binding sequence is the boxed segment at the 3′ end of the glmS ribozyme that forms a hybrid with the biotinylated ssDNA capture strand (orange). (b) Supernatants collected at various steps of the protocol and analyzed by denaturing, 8 M urea, 10% PAGE. “VS” refers to the targeted self-cleaved VS ribozyme, “glmS” to the self-cleaved glmS ribozyme with attached binding sequence, and “VS+glmS” to the transcript containing the VS ribozyme with attached uncleaved glmS ribozyme and binding sequence. (1.34 MB TIF) [file pone.0012953.s005.tif]

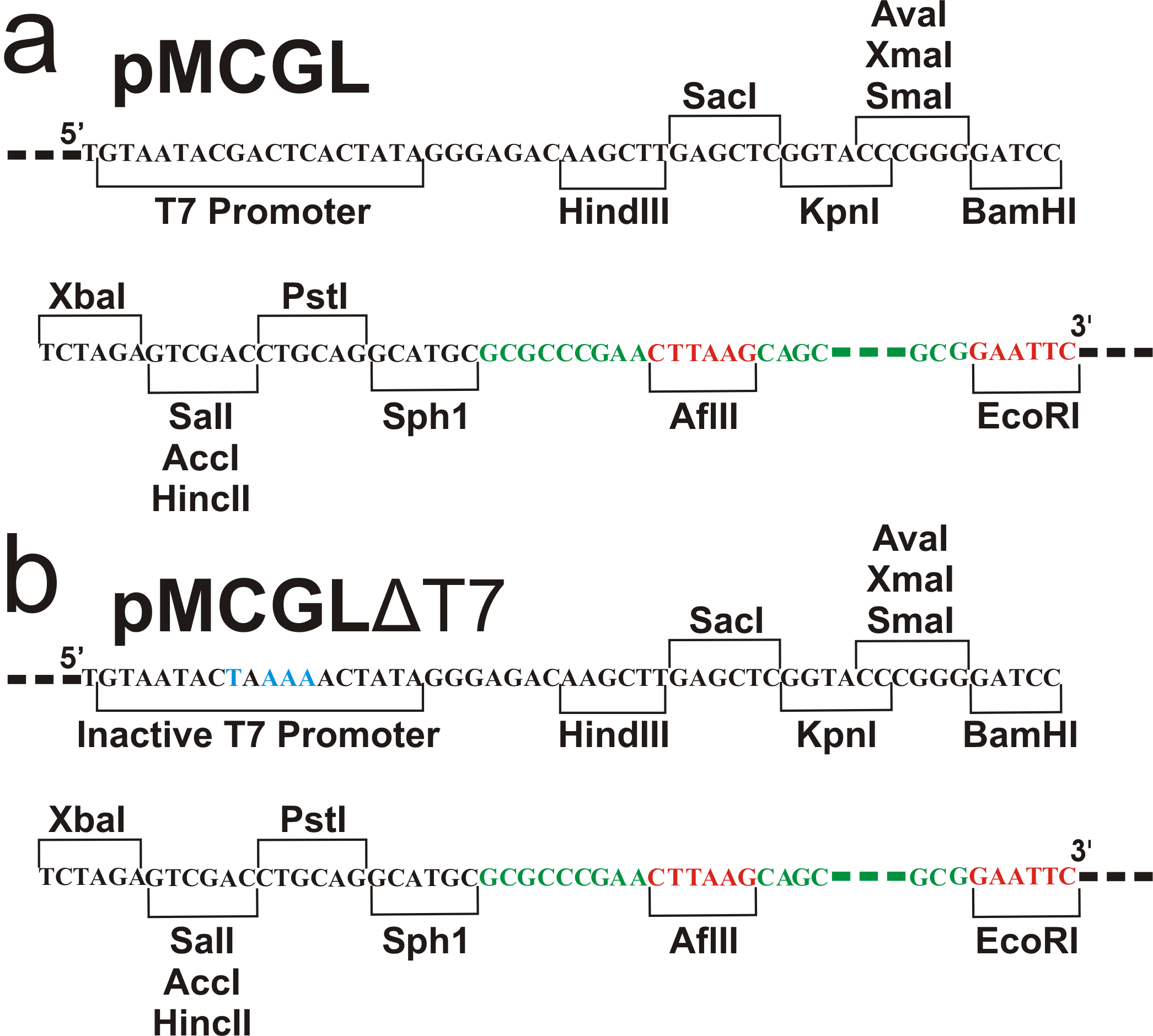

Supplement: Figure S6 — Sequences of the available plasmids with multiple cloning sites. The nucleotides and dotted line in green denote the sequence that encodes the glmS ribozyme. (a) The pMCGL plasmid contains an active T7 promoter upstream of the multiple cloning site. (b) In the pMCGLΔT7 plasmid the T7 promoter is inactivated by four point mutations (cyan). (0.61 MB TIF) [file pone.0012953.s006.tif]
